# Automated design of bacterial genome sequences

**DOI:** 10.1186/1752-0509-7-108

**Published:** 2013-10-25

**Authors:** Javier Carrera, Alfonso Jaramillo

**Affiliations:** 1 UC Davis Genome Center, University of California-Davis, Davis, CA, USA; 2 School of Life Sciences, University of Warwick, Gibbet Hill Road, Coventry CV4 7AL, UK; 3 Institute of Systems and Synthetic Biology, CNRS - Universite d'Evry Val d'Essonne. Batiment Geneavenir 6, 5 rue Henri Desbruères, Evry Cedex 91030, France

**Keywords:** Synthetic biology, Genome refactorization, Re-engineered genome nucleotide sequence

## Abstract

**Background:**

Organisms have evolved ways of regulating transcription to better adapt to varying environments. Could the current functional genomics data and models support the possibility of engineering a genome with completely rearranged gene organization while the cell maintains its behavior under environmental challenges? How would we proceed to design a full nucleotide sequence for such genomes?

**Results:**

As a first step towards answering such questions, recent work showed that it is possible to design alternative transcriptomic models showing the same behavior under environmental variations than the wild-type model. A second step would require providing evidence that it is possible to provide a nucleotide sequence for a genome encoding such transcriptional model. We used computational design techniques to design a rewired global transcriptional regulation of *Escherichia coli*, yet showing a similar transcriptomic response than the wild-type. Afterwards, we “compiled” the transcriptional networks into nucleotide sequences to obtain the final genome sequence. Our computational evolution procedure ensures that we can maintain the genotype-phenotype mapping during the rewiring of the regulatory network. We found that it is theoretically possible to reorganize *E. coli* genome into 86% fewer regulated operons. Such refactored genomes are constituted by operons that contain sets of genes sharing around the 60% of their biological functions and, if evolved under highly variable environmental conditions, have regulatory networks, which turn out to respond more than 20% faster to multiple external perturbations.

**Conclusions:**

This work provides the first algorithm for producing a genome sequence encoding a rewired transcriptional regulation with wild-type behavior under alternative environments.

## Background

The doors to new horizons in genome-scale synthetic biology have been opened by the recent and rapid development of technologies allowing the synthesis of novel genomes and their introduction into hosts with inactivated or deleted wild-type chromosomes [1,2]. The *de novo* design of cells with synthetic genomes that are viable in a well-defined environment might require only the constitutive expression of the minimal set of genes required for life [3]. This engineering approach, however, has several drawbacks, including the absence of all necessary blocks (e.g., genes, signaling cascades, etc.), the absence of a good definition of the minimal set of genes required, and a poor understanding of the pleiotropic negative effects that these genes may have when put together. In contrast, the re-engineering of an existing genome to change its regulation network would not require adding new genes to the genome but only their rearrangement with respect to promoter sequences. Previous work has considered the rearrangement of genomic sequences. For example, Chan *et al.* (2005) successfully modified the T7 genome to remove overlapping translational frames [4]. This approach was inspired by the engineering practice called refactoring, in which the internal structure of an already existing system is rearranged while its external function is maintained. Based on the same refactoring principle, and considering cell behavior as the “external function”, we have created a system for the design of a novel genome sequence with a refactored transcriptional regulatory network (TRN) that maintains its original behavior.

We ask whether is it possible to design cells with the same biochemical composition but different genetic information stored in the DNA. Because of our limitations in predicting phenotype from a genome, we restrict ourselves to the problem of rewiring transcriptional regulatory network to generate the same phenotype that the wild-type cell already has. Given the large number of gene regulations, it is not evident that such simplified question could be answered even based on theoretical considerations. Although the ultimate answer lies in the experimental verification, the development of a genomic-scale model showing the desired behavior is a necessary condition for such enterprise. In the context of synthetic biology [5], the design of an organism that can respond in a directed way to variations in its environment has been a particularly interesting and challenging problem. This design would require the reengineering of suitable signal transduction and regulation systems [6-9]. Because transcriptional regulation is the most well studied regulatory system in bacteria, it may be a good starting point for those interested in the design of such systems [10-12]. In fact, the recent experimental evolution of *E. coli* under changing environments has provided evidence of regulatory network rearrangements that allow anticipatory behavior [13]. However, the *de novo* design of a genome that can adapt to changing environments may be very challenging. A simpler alternative is to alter a pre-existing genome by reshuffling its genes in such a way that its behavior is maintained. In particular, this problem can be treated computationally if restricted to the re-design of the global transcriptional network for an organism for which sufficient transcriptomic information is available.

To evolve new genomes *in silico*, a necessary first condition is to define a biologically meaningful fitness function that allows changes that are introduced during the evolution process to be evaluated. How can such a fitness function be defined? We will assume that a given transcriptomic expression profile would determine the protein and metabolite concentrations of the cell, thus the biomass composition would ultimately result from the transcriptome. This can also be rationalized by arguing that natural selection results in nearly optimal biomass production by favoring regulation pathways that confer optimal levels of gene expression in a given environment. Consequently, we will construct a fitness function that will enforce the maintenance of the wild-type transcriptomic response. Like that, we would obtain the similar molecular composition in the cell under a given environment, while having a refactored genome. Interestingly, it has recently been shown that the transcriptomic expression profile is a good predictor of instantaneous cell growth in *Saccharomyces cerevisiae*[14]. Assuming that this relationship is true for other organisms, it can be hypothesized that the expression profile of a given system determines cell growth.

We can evaluate the validity of this hypothesis by analyzing the effect of mutations on the growth of a wild-type strain. Notably, this evaluation still requires the accurate prediction of a genome-scale expression profile. More modifications to the genome will lead to less growth and more differences in the expression profile. Therefore, we have used an automated methodology for designing a genome based on an *in silico* evolution process; the methodology uses similarity to a wild-type transcriptional profile as its fitness function, which provides the variation of cell growth. Furthermore, it is possible to construct regulatory network models that accurately predict the global transcriptional profile for some organisms [15,16]. These regulatory network models can be used to predict the growth of cells with modified transcriptional networks, thereby providing the fitness function required to evaluate their performance under diverse environmental conditions [17].

In this paper, we describe a methodology for generating nucleotide sequences of a genome that produce cells with targeted physiological responses to a set of environments. For this, we firstly use our previous integration of current known transcriptomic and signaling data into a global model consisting of differential equations, allowing the assignment of parameters to promoter and transcription factor (TF) coding sequences [17]. We begin the Results section by examining the outcome of this model construction and its corresponding properties. Next, we perform the computational design of the TRN with the aim of refactoring the *E. coli* regulation to simplify its internal structure by reducing the number of operons. Contrary to previous work [17], the rewiring of the TRN is done by ensuring the generation of a suitable nucleotide sequence. We found that we could dramatically reduce the number of operons while maintaining the organism’s response to fluctuating environments. We also analyzed other properties of the synthetic TRN, such as its topology and adaptation to varying environments. We then generated a genome sequence for the TRN. Finally, in the Discussion section, we examine some design principles that can be inferred from our results and future experimental applications of this work.

## Results and discussion

Our methodology to generate a refactorized genome sequence consists on three steps: *i*) reverse engineering the genomic transcriptional regulation network (GTRN) of wild-type *E. coli*, *ii*) the design of a rewired GTRN with the targeted behavior by evolutionary computation, and *iii*) the design of a nucleotide sequence for a genome implementing the GTRN. For the first step, we inferred the wild-type GTRN by using extensive transcriptomic and signaling data. For the second step, we use an evolutionary algorithm with a fitness function that would create a selection for transcriptomic profiles behaving as much as possible to the wild-type under selected environmental changes. Finally, we will create some rules that would allow choosing the appropriate genetic elements.

### GTRN of *E. coli*

**Figure 1:**
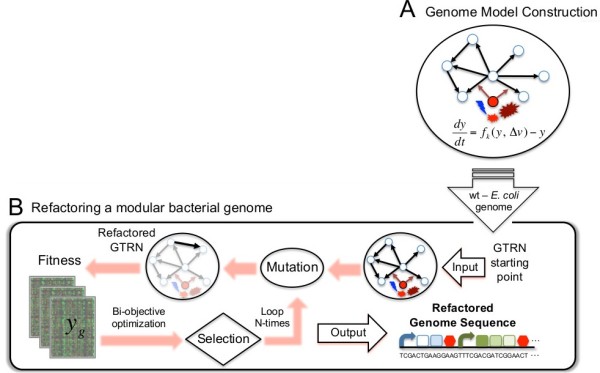
**Computational approach for the automated design of synthetic genome sequences. (A)** Steps designed to construct the regulatory network of E. coli required to sense environmental changes. **(B)** A scheme of the algorithm used to re-design the E. coli TRN [17]. The wild-type genome was used as the starting point for an optimization process based on Monte Carlo Simulated Annealing. During the in silico evolution, we modified gene regulation (Figure 2) and computed the resulting genome fitness as a function combining the genome modularity and the distance between the gene expression levels of the re-engineered and wild-type genomes.

**Figure 2:**
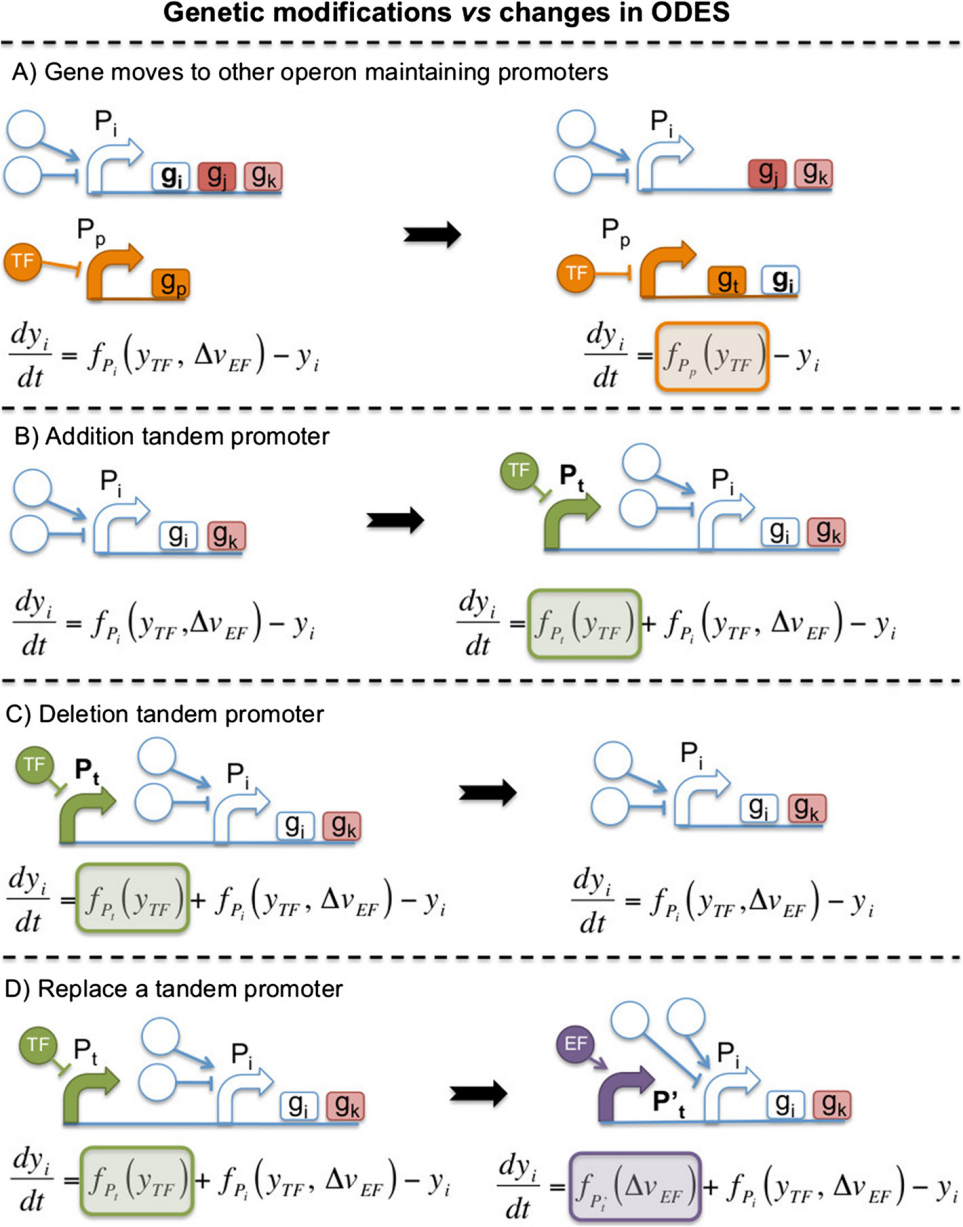
**Four types of transcriptional modifications during the optimization process that affect the gene expression of the ith gene. (A)** a gene moves to other operon, **(B and C)** addition or deletion of tandem promoters, and **(D)** replace a tandem promoter (see Methods section named “Automatic genome design: rules for mutation and selection”). All genetic perturbations are represented by the regulatory scheme with their corresponding ODE before (left) and after (right) the genome modification. Color boxes represent mathematical terms added or removed from the ODEs to simulate gene expression of the ith gene after the genetic modification.

We used a recent genome-wide model of *E. coli* gene transcription in response to selected external signals to predict changes in cell growth after genome modification [17]. Such model was inferred from experimental data and the, *InferGene* inference methodology [15], which is used to obtain kinetic parameters from experimental steady-state data. The model contains 4,298 non-redundant genes, 330 of which are putative TFs. As detailed in the Methods, this model is described by ordinary differential equations for the transcription level of each gene and its transcription regulation. This model allows the assignment of mathematical parameters to promoters and TF sequences, which we have assumed to be independent of genomic context (Figure 1A). In our previous work [17], we showed that we could predict experimental growth rates by assigning transcriptional parameters to genome regulatory sequences. Such assignment allows us to predict the TRN model after reshuffling genetic elements (Figure 2; see Methods).

### Evolutionary design of a rewired GTRN

**Figure 3:**
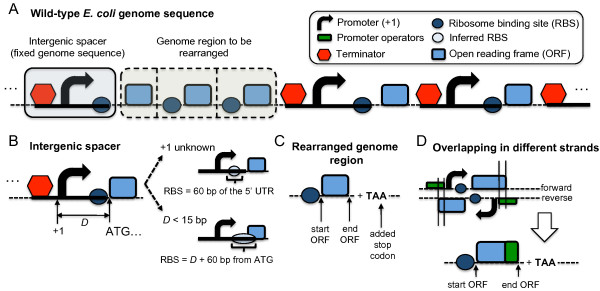
**Refactorization process of synthetic genome sequences. (A)** Wild-type E. coli genome sequence showing the fixed genome sequence and the regions to be rearranged during the refactorization. Genome region defined as intergenic spacer **(B)** and regions that can be rearranged **(C)**. Overlapping between a promoter region and an ORF **(D)**.

**Figure 4:**
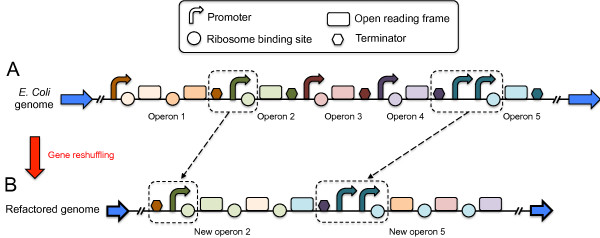
**Example of a refactored genome illustrating the gene rearrangement of 5 operons of the *E. coli *wild-type genome.** Note that blue arrows represent the rest of the wild-type **(A)** and refactored **(B)** genome.

Instead of trying to solve the challenging problem of evolving a genome for better growth, which would require a greater degree of accuracy for our fitness function, we attempted to reorganize the genome of *E. coli* while maintaining its functionality wild-type. We rearranged the TRN in terms of regulatory complexity and modularity. We applied our automatic design methodology [17] to perform the genome refactoring in a way that we could later provide a corresponding nucleotide sequence. This entails the rearrangement of the operon structure while maintaining the organism’s original behavior. We modify the GTRN by modifying the placement of genes and altering the promoter regulation, but we do it without loosing the connection with a nucleotide sequence. For instance, the operator sites of many promoter sequences are known, and their mutation to neutral sequences would presumably eliminate the regulation of the promoter. This genetic modification could be implemented in such a way that, once the optimal GTRN is found, we could design the suitable nucleotide sequence of such promoters (Figure 1B). The algorithm proceeds in successive rounds of genetic modifications and selection of a collection of independent GTRNs (typically 10 “cells” or simulations), a general strategy in evolutionary computation. Genetic modifications consist of the rearrangement of the certain genome regions of the nucleotide sequence of the wild-type genome, and selection is based on two criteria that determine the cellular growth rate and the modularity of the GTRN. Alternate phases of genetic modifications and selection are performed to evolve the GTRNs and obtain networks achieving the specified function. The mutation operator is conceived such that we could later produce the corresponding nucleotide sequence. According to the genome regions to be rearranged, some rules are described in Figure 3 to determine the rearranged nucleotide sequences (Figure 4).

### Methodology for producing a genome sequence for a GTRN

We will design now a genome sequence associated to a given GTRN (to “compile” the GTRN into a nucleotide sequence, using the computer analogy) by following an iterative procedure. We will start with the wild-type genome sequence, where a given base pair could belong to two possible types of genomic regions: the ones that will be kept fixed and those that could be rearranged (Figure 3A). For the former, we defined an intergenic spacer as a genome region that comprises three biological parts: *i*) a promoter region of a given operon, *ii*) a ribosome binding site (RBS) for the first cistron, and *iii*) the terminator region of the upstream operon. We choose to keep fixed the RBS sequence of the first gene of each operon because the 5’UTR sequence may not be well characterized and it may overlap with the promoter. To infer the RBS sequence, we considered the sequence fragment from the +1 position (transcription start) to the start codon. In some cases, the starting transcription is unknown and, consequently, we defined the RBS taking 60 base pairs (bp) from the 5’ UTR upstream the start codon. In addition, we also found 5’ UTRs in which the size of the nucleotide sequence was less than 15 bp and, to enlarge the RBS sequence, we included an additional 60 bp. Although leaderless translation is known to occur in *E. coli*[18], we still consider such 5’UTR. Although it is well known that the sequence and structure of the 5’UTR has a strong influence in translation initiation, we assume that the natural sequence upstream the start codon is already optimized for expression. When open reading frames (ORFs) move to other operons, they will loose their original RBS. As new RBS, we choose to add the same RBS sequence to any ORF arriving to the same destination operon. For this, we could either use a standardized RBS sequence or we could choose another sequence, such as the RBS from first ORF contained in that operon. In this later case, we append the inferred RBS sequence for the first ORF to each ORF of the subsequent cistrons (Figure 3B). Any other automated protocol to produce RBS sequences will also be limited in accuracy because we did not model the translation rates for our ORFs. By using the same RBS for every ORF in the destination operon, we better match our model assumptions (equal expression levels for every cistron). This is limited by the fact that i) ORFs downstream an operon will have lower translation levels and ii) we did not consider post-transcriptional regulation elements. Our algorithm could be extended to remove post-transcriptional elements by codon re-optimizing the ORFs and by using RBS without regulatory elements. In addition, we could extend our model by using high-throughput protein copy number data.

Then, we collected nucleotide sequences of all ORFs of *E. coli* genome by identifying their start and end from *RegulonDB* (version 5) [19]. We systematically added a stop codon (TAA) at the end of each ORF to define that genome region as a part of the genome susceptible to be rearranged, as a module, in another part of the genome (Figure 3C). In *RegulonDB*, we identified some overlaps between ORFs and promoter regions from operons located in different strands (Figure 3D). For those cases, we separated both operons to be rearranged considering that certain ORF sequences include small portions of promoter regions.

Figure 3 lists the possible genetic modifications, which consist of gene rearrangement (Figure 4) and addition/deletion/replacement of tandem promoters, and their translation into the equations. A gene can move to another operon (Figure 2A), downstream (upstream if in the reverse orientation) ORFs that did not move. This changes the equation for such gene to a new one with the regulatory function corresponding to the promoter of the arriving operon. This type of moves do not change very much the regulatory network and we added a genetic move that would construct a combinatorial promoter by appending two promoters in tandem (Figure 2B). This allows creating a much complex combinatorial regulation, without having a precise knowledge of the operator sites. We assume that it could always be possible to further optimize a suitable spacer to minimize promoter interference while having a 5’UTR for the transcript of the first promoter with similar translation initiation rates than the 5’UTR of the second promoter. Tandem promoter engineering has been used recently to design NOR gates [20]. We can also delete (Figure 2C) or replace (Figure 2D) a tandem promoter.

### Prediction of a refactored *E. coli* genome sequence with wild-type behavior in changing environments

**Figure 5:**
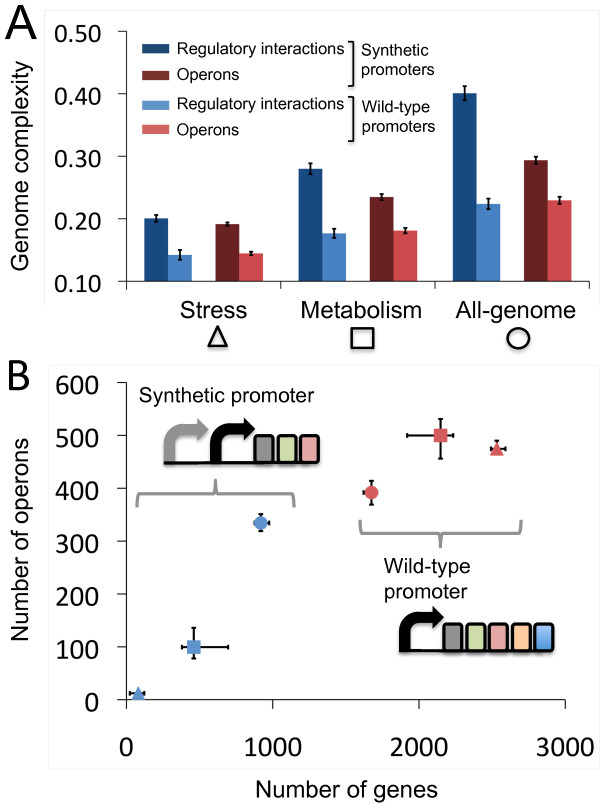
**Topological properties of refactored genomes. (A)** Complexity reduction (number of regulatory interactions and operons are represented using blue and red bars, respectively) of the refactored genomes with respect to the wild-type genome under permissive environments using an evolutionary process including tandem promoters (dark bars) or genetic re-organization of E. coli transcriptional units only (light bars). **(B)** Number of genes vs operons regulated by a given wild-type promoter (red points) or a tandem promoter added to the synthetic transcription unit (blue points), as proposed for the refactored genomes evolved using selective pressure based only on genes coding for enzymatic activity (squares), genes related to adaptation and defense functions (triangles) or the entire genome (circles). Error bars represent standard deviations of scores obtained from 10 independent.

We used the implemented evolutionary process to design refactored genomes containing only genetic building blocks that exist within the wild-type *E. coli* genome. The transcriptional regulation landscape that we explored contained all possible genome reconfigurations that could result from regrouping a set of genes under the control of a wild-type promoter. In Figure 5A, we observed a large reduction in the complexity of the refactored TRN quantified in terms of the ratio between the number of regulatory interactions (Ξ < 0.14; *p* < 0.001) and the number of operons (Θ < 0.14; *p* < 0.001) for the refactored and wild-type TRN, using a design function based on scoring the expression of stress genes (Methods). The nucleotide sequence of a refactored genome using this design function is provided in Additional file 1. Analogously, we found that limiting only the expression of genes coding for enzymes or genes related to defense and adaptation in the design produced larger reductions in complexity (Ξ < 0.18, Θ < 0.19 and Ξ < 0.23, Θ < 0.23, respectively; *p* < 0.001 in all cases).

**Figure 6:**
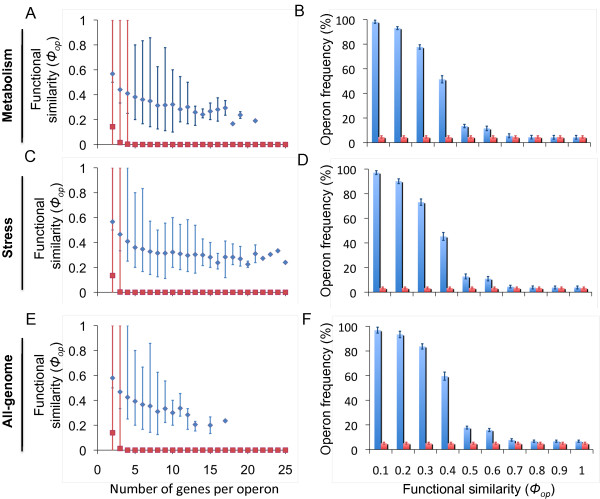
**Functional similarity (blue diamonds), depending on operon size, and a histogram showing the functional similarity (blue bars) of genes in the operons of genomes refactored under selective pressure on the expression of genes coding for enzymes (A and B, respectively), stress genes (C and D, respectively) and the whole genome (E and F, respectively).** Notice that red squares and bars represent random evolutionary processes. Error bars show the minimum and maximum value of functional similarity of all operons with a given size **(A, C and E)** and represent standard deviation of operon frequency **(B, D and F)** for 10 evolutionary processes.

To enlarge the genome design landscape, we allowed the addition of a maximum of three promoters in tandem to modify the regulation of a given operon (ethods). We determined a set of *E. coli* promoters that were potential candidates to operate in tandem (sometimes using a suitable spacer sequence to isolate them). We selected the entire promoter library (27 promoters) used by Isalan et al*.* (2008) to exhaustively explore the effect of multiple genome rewirings on growth rate [10]. We also included all *E. coli* promoters that are regulated by fewer than two master regulator TFs, as defined by Isalan et al*.* (2008). Consequently, we considered 272 promoters susceptible to tandem incorporation. Figure 5A shows that the largest reductions in complexity were achieved using designs that consider stress genes in the objective function (Ξ < 0.20, *p* < 0.001; Θ < 0.19, *p* < 0.001). Surprisingly, as shown in Figure 5B, few operons from the refactored genomes needed a promoter to be added in tandem to modify the gene expression provided by their wild-type promoter. Only 15 operons within the refactored genomes required the addition of two tandem promoters to guarantee that gene expression could adapt to changes in the environment. Such refactored genomes were characterized by operons that captured genes with similar functionality (Figure 6).

### Analysis of biochemical adaptation to varying environments of the refactored genome sequences

Two sets of environments were simulated to explore single environmental perturbations: (*i*) a set of 100 random perturbations that varied oxygen availability from a fully anaerobic environment to an environment with a rate that was 4-fold greater than the optimal flux value (75 mmol g^-1^ h^-1^) and (*ii*) a set of 100 perturbations that changed the availability of glucose as the carbon source, ranging from the negative value of the optimal uptake flux to the positive value (i.e., -20 mmol g^-1^ h^-1^ to 20 mmol g^-1^ h^-1^).

**Figure 7:**
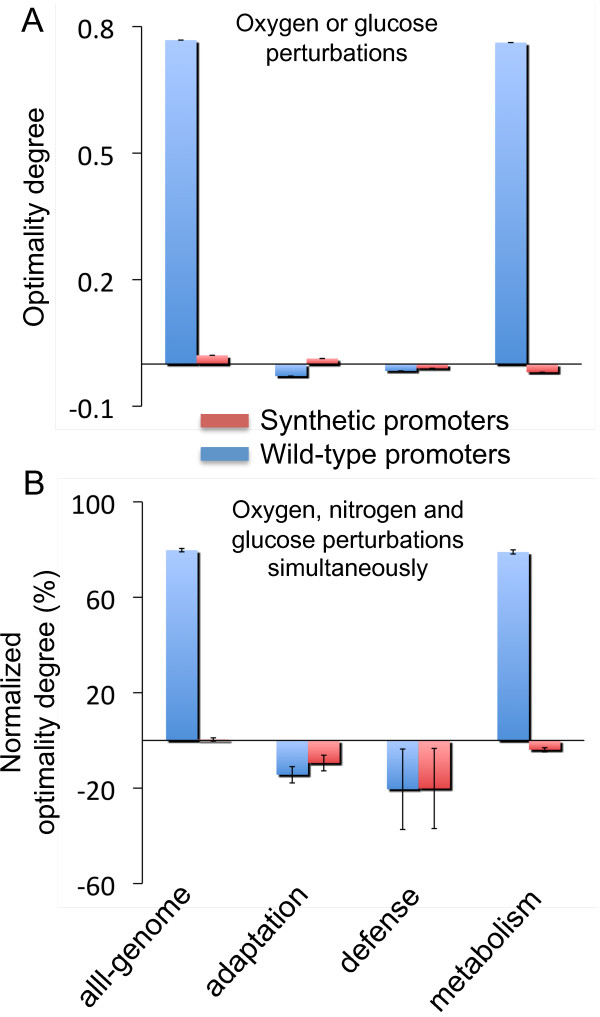
**Adaptive behavior of the refactored genomes evolved under different selective pressures (based on all genome or, stress, defense or metabolism related genes) in environments with single (A) or multiple (B) perturbations, respectively, with tandem promoters allowed (red bars) or not (blue bars).** Error bars represent standard deviations of the scores obtained from 10 evolutionary processes.

We tested adaptation in refactored genomes by considering the previous four types of selective pressure in the expression score. Interestingly, genomes that incorporated tandem promoters achieved low adaptation errors under single environmental perturbations (Figure 7A) (average optimality degree: 〈*ξ*〉 < 0.021) and over-optimality was even achieved by genomes designed with selection pressure based on genes with enzymatic activity (〈*ξ*〉 < -0.019) or related to defense processes (〈*ξ*〉 < -0.010). By contrast, genomes that were refactored without the design specification of tandem promoter addition had high error adaptation (〈*ξ*〉 > 0.762), except for those refactored considering stress genes. Furthermore, we tested the adaptation of genomes designed under multiple perturbations and concluded that evolved genomes that included tandem promoters exhibited over-optimality independent of the objective function imposed in the design. By contrast, genomes refactored by only re-organizing wild-type genes had adaptation errors as large as 〈*ξ*〉 = 79.8% (Figure 7B).

## Conclusions

### Biological consequences of computational genome refactorization

#### Genome organization can be simplified without disrupting the response of the genome to environmental changes

In this study, we have developed a computational framework for the design of bacterial genomes that are able to respond to changes in environmental conditions. We used transcriptomic data to infer a continuous model for the transcription of all *E. coli* genes [17], which we then used to assign appropriate parameters to promoter and TF coding sequences. By assuming that these parameters do not depend on genomic context in most cases, we proposed our first methodology for the automatic design of genome rearrangements under changing environments. Our results demonstrate that it is possible to refactorize the genome of *E. coli*, achieving an 86% reduction in the number of regulatory interactions and operons, while maintaining the ability to physiologically adapt to environmental changes. We found that the refactored genomes contain operons that encode several genes with similar functionality. This is an important result, given that the fitness function imposed to evaluate genome performance did not consider gene function. This agrees with the experimental observation that genes within an operon have similar functions [21]. Moreover, these genomes acquired the ability to adapt more rapidly to environmental changes, probably as a direct consequence of the reduced number of regulatory elements.

The refactored genomes satisfied the main design specification, which was to maintain the global physiological response under both optimal and changing environments. In addition, we found that there was an increase in the complexity of the internal structure related to the signal transduction for all refactored genomes. More specifically, genomes that evolved under the most extreme environ-ments required a greater re-organization of critical genes under promoters that could sense greater numbers of environmental interactions. Interestingly, genomes that were refactored under stressful environments showed higher clustering coefficients than those that evolved under more permissive environments. An intuitive explanation for this observation relies on the differences in the selective pressures imposed by both types of environments. Survival and replication in a stressful environment represents hard selection, requiring the coordinated expression of all genes involved in survival. By contrast, replicating in a permissive environment may be equated to soft selection and therefore does not require the coordination of expression because the cells remain able to exploit some components of their environment.

One important application of our results is the ability to infer some principles of genome design. In particular, we studied the refactored genomes that had achieved over-optimality or lost optimality. We tested the anticipatory ability of our refactored genomes by computing their optimality using transcriptomic fitness with the same set of genes used in the refactorization process. Interestingly, we found that refactored genomes achieved greater optimality degrees than those of wild-type genomes for both single and multiple environmental perturbations.

#### Extension of this methodology to other organisms

**Figure 8:**
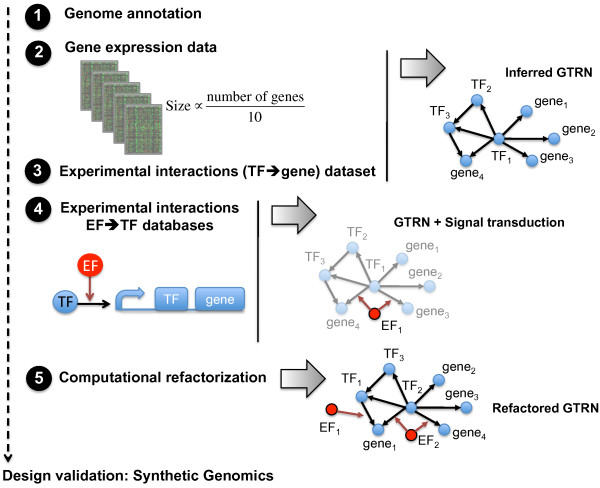
Requirements and extended methodology for the computational refactorization of an organism.

The methodology presented here could be extended to other organisms for which quantitative TRN and signal transduction models can be inferred (Figure 8) [22]. The models should be able to predict genomic transcriptional profiles under several external conditions in order to construct a transcriptomic fitness function. The computational refactorization of the genome of a given organism requires the following information: (*i*) genome annotation, (*ii*) a high-throughput gene expression data capturing genetic and environmental diversity, (*iii* and *iv*) datasets of transcriptional interactions (gene *vs.* TF) and two-component signal transduction pathways (TF *vs.* EF) that have been experimentally verified. Further extensions would have to consider the influence of other factors such as 3D localization [6], post-transcriptional regulation [23] and post-translational regulation in prokaryotes [24], or chromatin regulation [25] in eukaryotes. With growing availability of quantitative proteomics data [26], it will be pivotal to include variables representing protein copy number of the TFs into these models.

### Experimentally testable predictions

#### Proposition of a testable refactored E. coli genome sequence

This work also provides a generic procedure to generate a gene sequence for a synthetic *E. coli* genome with a targeted transcriptomic response, which we exemplify by proposing a genome that could be engineered by assembling known elements (see Additional file 1). For this quantitative prediction of a designed genome, we propose combining known promoter regions and transcriptional regulators such that the transcriptional profile could reasonably be predicted. To create more complex promoters, we propose taking advantage of their modularity and fusing some of them in tandem, and choosing a set of promoters for which transcriptional interference [27] would be minimized. Notice that the *E. coli* genome contains 166 non-overlapping tandem promoter pairs [27]. As sequence repetition could create ectopic recombination events, some care will have to be taken in experimental testing. In addition, wild-type transcription terminators are not completely efficient and are sometimes even absent; therefore, some terminators may have to be replaced by stronger ones (probably synthetic, to avoid repetitive sequences). The neglect of non-transcriptional regulation may produce unexpected behavior in certain environments, but this could be remedied by selecting alternative conditions. Other undesired behaviors could be alleviated by suitable randomization of the nucleotide sequence with the restriction of maintaining the desired functionality (e.g., the ribosome-binding site or protein coding sequence).

#### Application to experimental genome engineering

Our computational procedure could also be adapted to the particular needs of experimentalists who are willing to create major gene rearrangements in genomes using *in vivo* techniques. The recent advances in genome engineering (such as the use of the CRISPR technology) may enable synthetic biologists to produce large insertions, deletions or inversions in the genome [28]. Usually, targeted random mutagenesis followed by screening is used [29], but this methodology is tedious when a specific locus is to be targeted, and only a small number of successive modifications (*N*) would be practical. Therefore, it would be particularly useful for the genome engineer to know in advance the most suitable sequence of experiments to introduce genome modifications. In *E. coli*, this could be readily done by appropriately adapting the “mutational” moves used by our *in silico* evolution methodology. Such moves should be restricted to their genome rearrangement technologies available at the laboratory. Then, one could computationally explore all possible evolutionary paths of *N* moves that would give the highest fitness under specific dynamic environments. The experimentalist could then engineer the genome by implementing the *N* consecutive experiments suggested by the algorithm. This computational procedure could also incorporate the constraint that each intermediate genome should be viable.

## Methods

### Genome-scale model

We constructed a TRN of the wild-type genome that was able to predict gene regulation at the transcriptional and environmental levels. For this, we adopted a linear model based on differential equations describing the time dynamics of each mRNA [17] in order to infer real kinetic parameters for promoter and TF sequences. Thus, the mRNA dynamics from the *i*^th^ gene, *y*_*i*_, is given by *dy*_*i*_/*dt* = *α*_*i*_ + ∑ _*j*_* β*_* ij*_*y*_* j*_ + ∑ _*k*_*γ* _*ik*_*Δv*_*k*_ - *δ*_*i*_*y*_*i*_, where *a*_*i*_ represents its constitutive transcription rate, *b*_*ij*_ represents the regulatory effect that gene *j* has on gene *i*, *γ*_*ik*_ represents the effect that environmental factor (EF), i.e. the metabolic uptake factor *k*, has on the expression of gene *i*, *Δv*_*k*_ = ($v_{k}-v_{k}^{\mathrm{opt}}$) is the difference between the uptake factor measured under a given environmental condition, *v*_*k*_, and the uptake factor measured in the optimal environmental condition*,*$v_{k}^{\mathrm{opt}}$, and *d*_*i*_ represents the degradation and dilution rate constant (Figure 1A).

### Automatic genome design: fitness function

The main variables required for automatic genome design are the same as those required for any evolutionary algorithm [17]: (*i*) An initial genome, (*ii*) evolutionary steps represented by changes in the genome and (*iii*) a fitness function to evaluate the performance of each mutant genome (Figure 1B). For the first step, we used the genome of the model bacterium *E. coli*. The second step was achieved by dissecting the bacterial genome into elementary modules, to which evolutionary rules were applied [30]^.^

One design approach that we used involved the *in silico* refactorization of the nucleotide sequence of the *E. coli* genome, a process where we pursued two goals simultaneously: (*i*) simplifying the internal structure of *E. coli* and (*ii*) maintaining the external system function. To maximize the modularity of the system and thus simplify the TRN, we defined a measure based on the entropy of the genome [17]. We also aimed to maximize the similarity of the expression profiles of the wild-type and refactored genomes for a set of extreme environments and for a set of critical genes that guarantee the functionality of the refactored system. We used the TRN model integrated with signal transduction to measure that similarity.

### Automatic genome design: rules for mutation and selection

Considering these two aims, we developed an optimization algorithm based on the mutation rules to refactorize the wild-type *E. coli* genome (Figure 2 and 3). Genes that are controlled by constitutive promoters were not involved in the design. These genes could always be refactored in a straightforward way by assuming that they could be collapsed into large operons regulated by a gradient of different expression levels (produced by a library of several constitutive promoters or using tuned ribosome-binding sites).

Our algorithm searches possible reconfigurations of the global transcriptional regulation of *E. coli* such that the resulting modular genome contains all genes in a minimal set of operons, thus decreasing the number of transcriptional regulatory elements, and with the constraint that the overall gene expression of the refactored genome shall be as close to the wild-type as possible. We used Monte Carlo Simulated Annealing to perform the optimization in the space of all possible refactored transcriptional networks. The size of this combinatorial space is governed by the previously characterized variability in the *E. coli* natural promoters, and the diversity of synthetic promoters was obtained during the optimization process. As the starting condition, we assumed that the expression of each gene was controlled only by its natural promoter. The three following mutational steps were possible: (*i*) move gene *g*_*i*_ belonging to operon *op* and regulated by non-constitutive promoter *P*_*i*_ to another operon *op’* regulated by a different non-constitutive promoter *P*_*p*_ without adding regulatory operators to *P*_*p*_ (Figure 3A). We imposed the mathematical function from the promoter *P*_*p*_ (*f*_*p*_) in the ordinary differential equation (ODE) describing the expression of gene *g*_*i*_ (orange box in Figure 3A). (*ii*) Add or remove a promoter in tandem position, *P*_*t*_ (Figure 3B and C, respectively), in an operon (containing *g*_*i*_) controlled by the promoter *P*_*i*_. In terms of the set of ODEs describing gene expression, we add or remove the term *f*_*t*_ associated to the tandem promoter (green box in Figure 3B and C, respectively) in the ODE of gene *g*_*i*_. Promoters added in tandem to a given transcription unit could be removed or replaced by other promoters. Finally (*iii*) replace a promoter in tandem position (controlling gene expression of *g*_*i*_), *P*_*t*_, by another promoter suggested to act as a tandem promoter, *P*_*t’*_ (Figure 3D). In that way, we substituted the mathematical term associated to *P*_*t*_ (*f*_*t*_; green box in Figure 3D) by *P*_*t’*_ (*f*_*t’*_; purple box in Figure 3D). Note that the probability of removing/adding a promoter in tandem was set to be much larger than the probability of replacing one promoter in tandem with another promoter (e.g., 10-fold).

Then, we simulate the expression behavior of the newly created genome and compute its new objective function (*S*_*new*_), which depends on the full transcriptome predicted under a set of environments and the new modular organization of the operons. If the suggested mutation improves *S* (*S*_*new*_ ≥ *S*), then it is accepted. Otherwise, it is accepted with probability $e^{\left(S-S_{\mathrm{new}}\right)/T}$, where *T* is a Boltzmann temperature parameter that decreases exponentially with the number of iterations. Hereafter, we loop back and introduce a new transcriptional modification.

### Genome optimality degree in changing environments and functional analysis of refactored genomes

We assumed that cell fitness could be estimated in terms of the *S*_*exp*_ objective function. This allowed the study of genome adaptation under changing environments in one (*Δv*_*k* = *i*_ ≠ 0 and *Δv*_*k* ≠ *i*_ = 0) or multiple (*Δv*_*k*_ ≠ 0 ∀ *k*) directions [31]. To do this, we defined the optimality degree, $\xi_{\Delta v_{k}}$, in a target environment characterized by *Δv*^∗^_*k*_ and different from the optimal environment as the difference between *S*_exp_ evaluated in an environment containing *Δv*_*k*_ = 0 (i.e., fitness in the optimal condition) and that evaluated in the target environment containing *Δv*^∗^_*k*_. Hence, we distinguished between positive and negative error adaptation corresponding to environmental states where cell fitness achieved sub- or over-optimal growth, respectively.

Genes contained in the operons of all refactored genomes were functionally identified using 184 biological functions in GO [32]. We defined the degree of functional similarity, *Φ*_*op*_, of a given operon, *op*, as the ratio between the maximum number of genes with the same functionality and the operon size. We imposed *Φ*_*op*_ = 0 for those operons containing only one gene because more than one gene was needed to assess functional similarity; all operons in the wild-type genome therefore received a score of 0.

## Abbreviations

TF: Transcription factor; ODE: Ordinary differential equation; TRN: Transcriptional regulatory network; GTRN: Genomic transcriptional regulatory network; ORF: Open reading frame; RBS: Ribosome binding site.

## Competing interests

The authors declare that they have no competing interests.

## Authors’contributions

AJ conceived the study. JC and AJ performed all the computations, analyzed the data and contributed to writing the manuscript. All authors read and approved the final manuscript.

## Supplementary Material

Additional file 1 **It includes three files: Nucleotide sequence (FASTA and GeneBank files) and SBML model for the refactored *E. coli *genome shown in Figure** 5**A (non tandem promoter addition (light bars) and, fitness based on the set of stress-related genes).** Click here for file
